# Similar clinical outcomes with transcatheter aortic valve implantation and surgical aortic valve replacement in octogenarians with aortic stenosis

**DOI:** 10.3389/fcvm.2022.947197

**Published:** 2022-10-25

**Authors:** Tadeja Kolar, Nikola Lakič, Aleša Kotnik, David Štubljar, Zlatko Fras, Matjaž Bunc

**Affiliations:** ^1^Department of Cardiovascular Surgery, University Medical Center Ljubljana, Ljubljana, Slovenia; ^2^Medical Faculty, University of Ljubljana, Ljubljana, Slovenia; ^3^In-Medico, Department of Research and Development, Metlika, Slovenia; ^4^Department of Cardiology, University Medical Centre Ljubljana, Ljubljana, Slovenia

**Keywords:** TAVI, SAVR, octogenarians, aortic valve stenosis, aortic valve replacement

## Abstract

**Background:**

Transcatheter aortic valve implantation (TAVI) is the preferred treatment option for severe aortic stenosis in the elderly and in patients with comorbidities. We sought to compare outcomes after TAVI and surgical aortic valve replacement (SAVR) in octogenarians.

**Methods:**

In this retrospective cohort study conducted at our tertiary center, clinical data were gathered before and after TAVI and SAVR procedures performed from January 2013 to May 2019; follow-up completed in March 2021. The primary outcome was 1-year mortality. Patients were stratified according to Society of Thoracic Surgeons (STS) score and procedure type. Propensity score-based matching was also performed.

**Results:**

Of 542 patients who matched the inclusion criteria, 273 underwent TAVI and 269 SAVR. TAVI patients were older (85.8 ± 3.0 vs. 82.2 ± 2.2 years; *P* < 0.001) and had a higher mean STS score (5.0 ± 4.0 vs. 2.8 ± 1.3; *P* < 0.001) and EuroSCORE II (5.3 ± 4.1 vs. 2.8 ± 6.0; *P* < 0.001). Rates of postoperative permanent pacemaker insertion (15.0% vs. 9.3%; *P* = 0.040) and paravalvular leak (9.9% vs. 0.8%; *P* < 0.001) were higher and acute kidney injury lower (8.8% vs. 32.7%; *P* < 0.001) after TAVI, with no difference between treatment groups for major bleeding (11.0% vs. 6.7%; *P* = 0.130) or 30-day mortality (5.5% vs. 3.7%; *P* = 0.315). A statistically significant difference was found between TAVI and SAVR in low- and intermediate-risk groups when it came to occurrence of paravalvular leak, acute kidney injury, and new onset AF (all *P* < 0.001).

**Conclusion:**

This analysis of an octogenarian “real-life” population undergoing TAVI or SAVR (with a biological valve) showed similar outcomes regarding clinical endpoints in low- and medium-risk (STS score) groups.

## Introduction

Aortic stenosis (AS) is a common, progressive valvular lesion with a poor prognosis when left untreated (1). Octogenarian patients with AS have a high prevalence of coexisting conditions and may be at increased risk of periprocedural morbidity and mortality when undergoing surgery (2).

Surgical aortic valve replacement (SAVR), first performed in the 1960s, became the gold standard for treating AS (3), but surgeons were initially reluctant to operate on older patients (2). Minimally invasive SAVR, a modification of the original technique, was developed with the purpose of minimizing operative trauma and reducing postoperative mortality and morbidity (4). Balloon valvotomy was the first procedure to emerge as a possible endovascular treatment (3). Aspiration, an even less-invasive method—suitable for patients with little or no chance of surviving surgery—led to implantation of the first transcatheter aortic valve implantation (TAVI), in 2002. Since then, TAVI for AS has been increasingly adopted in clinical practice (5).

With well-established surgical methods it was not easy to prove the non-inferiority of TAVI (6). However, with meticulously designed studies conducted over the past decade, advocates of TAVI not only demonstrated its non-inferiority in high-risk patients, they proved its benefits, and expanded its indication for use in intermediate- and low-risk patients (7–11).

Advances in treatment techniques have led to different options to consider for our patients. The first mention of TAVI was in the 2008 guidelines on the treatment of AS. More recently, evidence from clinical trials has now positioned TAVI as a suitable treatment option for AS (12–14).

The objective of this study was to compare clinical outcomes among octogenarian patients undergoing TAVI or SAVR treated at our facility during the past decade.

## Materials and methods

### Patients and data source

In this retrospective cohort study, data from two local registries of patients who had undergone TAVI or SAVR for severe AS at the University Clinical Centre in Ljubljana between January 2013 and May 2019 were gathered. During that period, 3,384 patients had undergone surgical treatment and 513 had undergone TAVI. Our search was restricted to patients older than 80 years at the time of the procedure who underwent isolated treatment of AS. The surgical group included patients who had a biological valve implanted. Additional exclusion criteria were active infective endocarditis, reoperation, and valve-in-valve TAVI. Follow-up data were retrieved by reviewing hospital records and national registries.

The National Ethical Committee approved the study design. Informed consent was waived due to the retrospective nature of the study.

### Treatment approach

In the TAVI group, the Sapien XT (Edwards Lifesciences, Irvine, CA), Sapien 3 (Edwards Lifesciences), Evolut R (Medtronic, Minneapolis, MN, USA), and Portico (St Jude Medical, Austin, TX) valves were used. The decision on the type of procedure was made by the interdisciplinary heart team. In the SAVR group, the choice of procedure and type of valve were at the surgeon’s discretion. All patients received sutureless or stented biological valves [Trifecta (Abbott, St. Paul, Minnesota, USA), Magna (Edwards Lifesciences), Mitroflow (Sorin Group, Inc., Milan, Italy), Epic (Abbott), Freedom solo (Sorin Group, Inc.), Enable (ATS Medical, Minneapolis, MN), Intuity (Edwards Lifesciences), Perceval (Sorin Group, Inc.), Crown (Sorin Group, Inc.)].

All patients were preoperatively assessed and evaluated with using validated scoring systems, keeping in mind patient frailty and comorbid conditions. High-risk patients were initially defined as those with a logistic EuroSCORE ≥ 20 or a EuroSCORE II ≥ 7, and later as a Society of Thoracic Surgeons Predicted Risk of Mortality (STS-PROM) score ≥ 8.0, or using other criteria not included in the scoring systems (patient frailty, porcelain aorta, movement impairment, patient request etc.). At first only patients declined for SAVR were selected for TAVI. From 2017, with the revision of guidelines, the criteria were adjusted according to the new findings, and the multidisciplinary heart team (interventional cardiologist, cardiac surgeon, cardiologist) was involved in decision making process for each patient with severe aortic stenosis requiring a form of treatment.

### Clinical outcomes

The primary outcome was 1-year mortality. Secondary outcomes were post-procedural acute kidney injury, new onset atrial fibrillation (AF) (≤30 days), permanent pacemaker implantation (PPI), cerebrovascular stroke or transient ischemic attack (TIA), paravalvular leak, major or life-threatening bleeding, 30-day mortality, in-hospital mortality, and length of hospital stay. All outcomes were defined according to the Valve Academic Research Consortium-2 criteria (15).

### Statistical analysis

Categorical variables are presented as frequencies with percentages, and continuous variables as mean values with standard deviations (SD). Patient death was evaluated as the dependent variable. Normally distributed quantitative variables were analyzed using the one-way ANOVA test, and abnormally distributed variables using the non-parametric Kruskal-Wallis test. Qualitative data were compared using Pearson’s χ^2^-test, where the statistical difference between the independent variables and the variable was determined. Cox regression and Kaplan-Meier survival curves were used to assess the probability for death. A two-sided *P*-value less than 0.05 was considered to indicate statistical significance. Nearest neighbor propensity score matching (PSM) with ratio of 1:1 and caliper width set at 0.2 of the standard deviation of logit of propensity score was performed using the MatchIt package in R (Language and environment for statistical computing. R Foundation for Statistical Computing, Vienna, Austria). Balance between the two groups was maintained by keeping standardized mean difference less than 0.15 and variance ratios between 0.5 and 2. All analyses were performed using SPSS 21 software (IBM, New York, USA).

## Results

### Study population and operative characteristics

**FIGURE 1:**
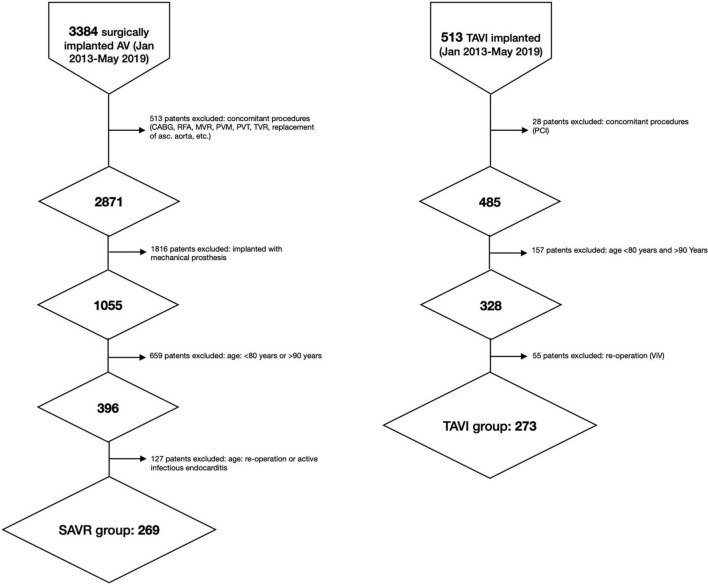
Flow-chart. asc, ascending; AV, aortic valve; CABG, coronary artery bypass graft; RFA, radiofrequency ablation; MVR, mitral valve replacement; PCI, percutaneous coronary intervention; PVT, plasty of tricuspid valve, PVM, plasty of mitral valve; TVR, tricuspid valve replacement; SAVR, surgical aortic valve replacement; TAVI, transcatheter aortic valve implantation.

The study population included detailed information on 542 patients: 273 (50.4%) underwent TAVI and 269 (49.6%) SAVR (Figure 1). Follow-up was completed in March 2021. Patients in the TAVI group were older than those in the SAVR group and had a higher mean EuroSCORE II and STS score (all *P* < 0.001) (Table 1).

**TABLE 1:** Patient baseline characteristics.

	Overall			PSM		
		
	TAVI *n* = 273	SAVR *n* = 269	*P*-value	TAVI *n* = 85	SAVR *n* = 85	*P*-value
Age in years, mean ± SD	85.8 ± 3.0	82.2 ± 2.2	<0.001	84.1 ± 2.4	84.2 ± 2.5	0.740
Male sex, *n* (%)	97 (35.5)	102 (37.9)	0.564	27 (31.8)	30 (35.3)	0.626
Body mass index in kg/m^2^, mean ± SD	26.6 ± 4.3	26.6 ± 3.8	0.987	26.5 ± 3.9	27.1 ± 4.0	0.353
Arterial hypertension, *n* (%)	256 (93.8)	250 (92.9)	0.696	82 (96.5)	82 (96.5)	1.000
Diabetes mellitus, *n* (%)	69 (25.3)	55 (20.4)	0.181	19 (22.4)	18 (21.2)	0.853
Coronary artery disease, *n* (%)	101 (37)	55 (20.4)	<0.001	27 (31.8)	20 (23.5)	0.230
Recent MI (≤30 days), *n* (%)	14 (5.1)	2 (0.7)	0.003	2 (2.4)	2 (2.4)	1.000
Peripheral vascular disease, *n* (%)	67 (24.5)	14 (5.2)	<0.001	9 (10.6)	12 (14.1)	0.484
Syncope, *n* (%)	46 (16.8)	49 (18.2)	0.676	16 (18.8)	14 (16.5)	0.687
Atrial fibrillation, *n* (%)	126 (46.1)	69 (25.7)	<0.001	32 (37.6)	33 (38.8)	0.875
Ejection fraction, *n* (%)			0.008			0.457
>50%	198 (72.5)	223 (82.9)		70 (82.4)	67 (78.8)	
30-50%	59 (21.6)	38 (14.1)		14 (16.5)	18 (21.2)	
<30%	6 (2.2)	1 (0.4)		1 (1.2)	0	
No data available	10 (3.7)	7 (2.6)		–	–	
Pulmonary hypertension, *n* (%)	188 (68.9)	119 (44.2)	<0.001	54 (63.5)	52 (61.2)	0.563
NYHA class, *n* (%)			<0.001			0.112
I	2 (0.7)	30 (11.2)		2 (2.4)	6 (7.1)	
II	93 (34.1)	143 (53.2)		38 (44.7)	40 (47.1)	
III	170 (62.3)	85 (31.6)		44 (51.8)	34 (40.0)	
IV	8 (2.9)	9 (3.3)		1 (1.2)	5 (5.9)	
No data available	0	2 (0.7)				
Chronic pulmonary disease, *n* (%)	29 (10.6)	19 (7.1)	0.145	9 (10.6)	9 (10.6)	1.000
Previous cerebrovascular stroke or TIA, *n* (%)	6 (2.2)	1 (0.4)	0.103	1 (1.2)	1 (1.2)	1.000
Tumor, *n* (%)	33 (12.1)	23 (8.6)	0.176	6 (7.1)	7 (8.2)	0.773
PCI < 3 months before the procedure, *n* (%)	20 (7.3)	3 (1.1)	<0.001	5 (5.9)	3 (3.5)	0.469
Preoperative creatinine in μ mol/l, mean ± SD	101.0 ± 51.7	86.9 ± 25.6	<0.001	91.9 ± 41.3	92.6 ± 28.0	0.895
Preoperative hemoglobin in g/l, mean ± SD	123.8 ± 16.1	128.0 ± 13.9	0.001	125.5 ± 18.1	126.4 ± 12.9	0.696
STS score, mean ± SD	5.0 ± 4.0	2.8 ± 1.3	<0.001	3.8 ± 2.2	3.3 ± 1.6	0.090
EuroSCORE II, mean ± SD	5.3 ± 4.1	2.8 ± 6.0	<0.001	3.9 ± 2.1	3.9 ± 1.4	0.998

MI, myocardial infarction; NYHA, New York Heart Association; PCI, percutaneous coronary intervention; SAVR, surgical aortic valve replacement; SD, standard deviation; STS, Society of Thoracic Surgeons; TAVI, transcatheter aortic valve implantation; TIA, transient ischemic attack, PSM, propensity score matched.

Operative characteristics are summarized in Table 2. In the TAVI group, 87.9% of patients had transfemoral TAVI, 6.2% had transapical TAVI, and 5.9% had transaortic TAVI. Of the patients undergoing SAVR, 76.6% had minimally invasive SAVR and 23.4% underwent full sternotomy.

**TABLE 2:** Perioperative data according to TAVI or SAVR.

	TAVI *n* = 273	PSM TAVI *n* = 85
**Procedure type, *n* (%)**		
Transfemoral	240 (87.9)	80 (94.1)
Transapical	17 (6.2)	2 (2.4)
Transaortic	16 (5.9)	3 (3.5)
**Valve implanted, *n* (%)**		
Edwards Sapien XT	72 (26.4)	17 (20.0)
Edwards Sapien 3	54 (19.8)	23 (27.1)
Medtronic Evolut R	133 (48.7)	39 (45.9)
St Jude Medical Portico	10 (3.7)	4 (4.7)
Other	4 (1.5)	2 (2.4)

	**SAVR *n* = 269**	**PSM SAVR *n* = 85**

**Procedure type, *n* (%)**		
Full sternotomy	63 (23.4)	19 (22.4)
Mini J sternotomy	103 (38.3)	30 (35.3)
Mini right thoracotomy	103 (38.3)	36 (42.3)
**Valve implanted, *n* (%)**		
SJM Trifecta	52 (19.3)	17 (20.0)
Medtronic enable	38 (14.1)	13 (15.3)
Sorin perceval	120 (44.6)	41 (48.2)
Sorin Freedom Solo	18 (6.7)	6 (7.1)
Edwards Intuity	28 (10.4)	5 (5.9)
Other	13 (4.8)	3 (3.5)

SAVR, surgical aortic valve replacement; TAVI, transcatheter aortic valve implantation; PSM, propensity score matched.

After performing propensity score analysis for our population, demographic, and clinical characteristics became well balanced between 170 matched patients. The matched population clinical and procedural characteristics are shown in Tables 1, 2.

### Clinical outcomes

Clinical outcomes according to TAVI or SAVR are detailed in Table 3. Mean follow-up was 45.2 ± 25.6 months; 3 patients (0.5%) were lost to survival follow-up.

**TABLE 3:** Clinical outcomes according to TAVI or SAVR.

	Overall			PSM		
		
	TAVI *n* = 273	SAVR *n* = 269	*P*-value	TAVI *n* = 85	SAVR *n* = 85	*P*-value
Acute kidney injury, *n* (%)	24 (8.8)	88 (32.7)	<0.001	5 (5.9)	12 (14.1)	<0.001
New-onset atrial fibrillation, *n* (%)	12 (4.4)	82 (30.5)	<0.001	0	23 (27.1)	<0.001
Cerebrovascular stroke or TIA, *n* (%)	2 (0.7)	8 (3.0)	0.053	0	2 (2.4)	0.155
Permanent pacemaker implantation, *n* (%)	41 (15.0)	25 (9.3)	0.040	12 (14.1)	4 (4.7)	0.036
Paravalvular leak, *n* (%)			<0.001			<0.001
Trivial or no leak	101 (37.0)	217 (80.7)		22 (25.9)	65 (76.5)	
Mild	125 (45.8)	13 (4.8)		50 (58.8)	3 (3.5)	
Moderate	26 (9.5)	1 (0.4)		11 (12.4)	1 (1.2)	
Severe	1 (0.4)	1 (0.4)		0	0	
No data available	20 (7.3)	37 (13.8)		2 (2.3)	16 (18.8)	
Major bleeding, *n* (%)	30 (11.0)	18 (6.7)	0.130	7 (8.2)	9 (10.5)	0.599
Hospital stay in days, mean ± SD	9.8 ± 6.1	10.8 ± 6.8	0.657	8.9 ± 4.9	10.8 ± 6.6	0.032
In-hospital mortality, *n* (%)	9 (3.3)	8 (3.0)	0.829	1 (1.2)	8 (9.4)	0.016
30-day mortality, *n* (%)	15 (5.5)	10 (3.7)	0.315	1 (1.2)	9 (10.6)	0.009
1-year mortality, *n* (%)	28 (10.3)	18 (6.7)	0.126	6 (5.9)	13 (15.3)	0.088

SAVR, surgical aortic valve replacement; TAVI, transcatheter aortic valve implantation; TIA, transient ischemic attack, PSM, propensity score matched.

TAVI was associated with a lower rate of new-onset AF and acute kidney injury (both *P* < 0.001). More than 40% of patients had acute kidney injury after transapical TAVI compared with only 6.7% among those who had transfemoral TAVI. In the SAVR group, patients with an aortic cross-clamp time exceeding 50 min had a 50% higher occurrence of acute kidney injury.

The rate of PPI (*P* = 0.040) and the number of patients with mild-to-severe paravalvular leak (*P* < 0.001) were higher in the TAVI group. Cerebrovascular stroke or TIA was reported in 2 patients (0.7%) in the TAVI group and in 8 patients (3.0%) in the SAVR group (*P* = 0.053). Major bleeding occurred in 11.0% of patients following TAVI and in 6.7% after SAVR, but the difference was not statistically significant (*P* = 0.130). There were no differences between TAVI and SAVR groups for length of hospital stay, in-hospital mortality, 30-day mortality, or 1-year mortality.

Propensity score analysis for our population showed similar clinical outcomes with the exception of length of hospital stay (*P* = 0.032), in hospital mortality (*P* = 0.016) and 30-day mortality (*P* = 0.009), all in favor of TAVI group (Table 3).

The results of a Cox regression analysis predicting mortality at 30 days and 1 year are shown in Table 4. Recent myocardial infarction (within 1 month before the procedure) and STS-PROM score were identified as significant predictors of death at 30 days in both groups. In the TAVI group, STS-PROM score, recent myocardial infarction, and creatine concentration were predictive of death at 1 year. In the SAVR group, only recent myocardial infarction was a predictor of death at 1 year.

**TABLE 4:** Predictors of mortality at 30 days and 1 year (Cox regression).

Mortality	All patients			TAVI			SAVR		
			
At 30 days	B	*P*-value	HR (95% CI)	B	*P*-value	HR (95% CI)	B	*P*-value	HR (95% CI)
Recent MI (≤ 30 days)	1.994	0.000	7.345 (2.518-21.426)	1.693	0.009	5.437 (1.532-19.291)	2.862	0.007	17.505 (2.203-139.110)
New onset atrial fibrillation	0.166	0.685	1.180 (0.530-2.627)	–0.272	0.605	0.762 (0.271-2.140)	0.666	0.302	1.946 (0.549-6.898)
Diabetes mellitus	0.278	0.533	1.320 (0.551-3.161)	0.072	0.902	1.075 (0.342-3.375)	0.522	0.449	1.686 (0.436-6.521)
Arterial hypertension	0.540	0.597	1.716 (0.232-12.681)	3.100	0.508	22.191 (0.002-214,356.258)	–0.389	0.712	0.678 (0.086-5.351)
Creatinine concentration (per increase of 1 μmol/l)	0.004	0.190	1.004 (0.998-1.010)	0.004	0.171	1.004 (0.988-1.010)	–0.007	0.596	0.993 (0.966-1.020)
STS-PROM (per increase of 1 UNIT)	0.072	0.001	1.075 (1.030-1.121)	0.065	0.009	1.067 (1.016-1.120)	0.342	0.044	1.408 (1.010-1.964)
**At 1 year**									
Recent MI (≤ 30 days)	1.598	0.000	4.905 (2.108-11.413)	1.427	0.003	4.165 (1.610-10.771)	2.101	0.040	8.178 (1.104-60.579)
New onset atrial fibrillation	0.318	0.222	1.374 (0.825-2.290)	0.010	0.977	1.010 (0.515-1.980)	0.641	0.112	1.898 (0.861-4.183)
Diabetes mellitus	0.557	0.042	1.754 (1.020-2.984)	0.371	0.312	1.449 (0.706-2.972)	0.761	0.065	2.141 (0.954-4.804)
Arterial hypertension	–0.009	0.987	0.992 (0.360-2.734)	0.828	0.415	2.289 (0.313-16.735)	–0.594	0.333	0.552 (0.166-1.840)
Creatinine concentration (per increase of 1 μmol/l)	0.004	0.024	1.004 (1.001-1.008)	0.004	0.042	1.004 (1.000-1.008)	0.003	0.724	1.003 (0.988-1.017)
STS-PROM (per increase of 1 UNIT)	0.064	0.005	1.066 (1.020-1.114)	0.053	0.048	1.055 (1.000-1.112)	0.215	0.071	1.248 (0.982-1.566)

B, correlation coefficient; CI, confidence interval; HR, hazard ratio; MI, myocardial infarction; SAVR, surgical aortic valve replacement; TAVI, transcatheter aortic valve insertion; STS-PROM, Society of Thoracic Surgeons predicted risk of mortality.

**FIGURE 2:**
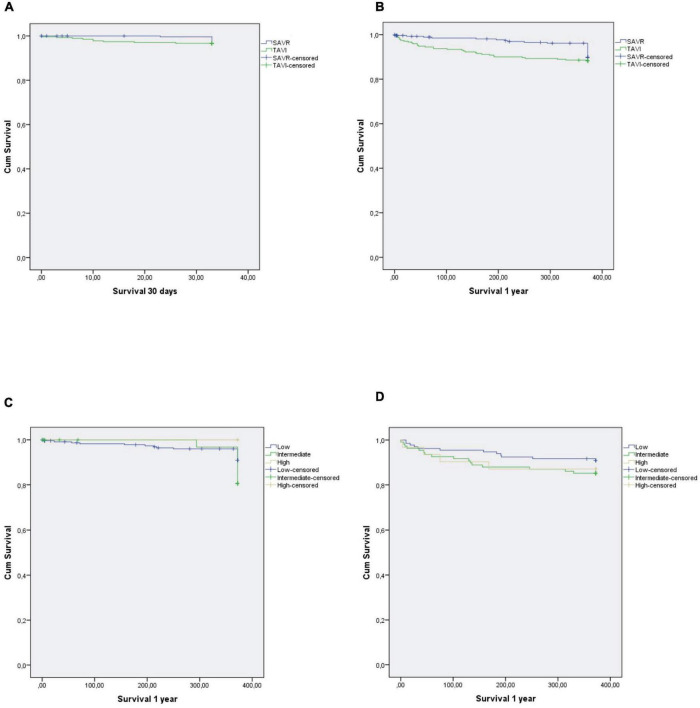
Kaplan-Meier survival curves according to TAVI or SAVR: **(A)** SAVR vs. TAVI—30-day survival, **(B)** SAVR vs. TAVI—1-year survival, **(C)** SAVR—1-year survival stratified by STS score, **(D)** TAVI—1-year survival stratified by STS score; SAVR, surgical aortic valve replacement; STS, Society of Thoracic Surgeons; TAVI, transcatheter aortic valve implantation.

Stratification of postoperative results by STS score and procedure (TAVI vs. SAVR: low risk: 48.3% vs. 85.5%; intermediate risk: 39.6% vs. 13.4%; high risk: 12.1% vs. 0.7%) is detailed in Table 5. When comparing groups, a statistically significant difference was found between TAVI and SAVR in low- and intermediate-risk groups when it came to occurrence of paravalvular leak, acute kidney injury, and new onset AF (all *P* < 0.001). Kaplan-Meier survival curves showed no differences between treatment groups (30 days: 3.7% vs. 5.5%, *P* = 0.315; 1 year: 6.7% vs. 10.2%, *P* = 0.126; respectively) (Figure 2).

**TABLE 5:** Outcomes after STS stratification according to TAVI or SAVR.

STS	TAVI, %			SAVR, %			*P*-value (comparing STS risk groups)			*P*-value (between treatment groups)	
				
	<4 *n* = 132	4-8 *n* = 108	>8 *n* = 33	<4 *n* = 230	4-8 *n* = 36	>8 *n* = 2	<4	4-8	>8	SAVR	TAVI
1-year mortality	9.8	15.7	12.9	8.7	16.7	0	0.714	0.896	0.588	0.290	0.390
30-day mortality	3.8	7.4	6.4	2.6	11.1	0	0.529	0.486	0.711	0.042	0.462
Cerebrovascular stroke or TIA	1.5	0	0	3.0	2.8	0	0.369	0.084	–	0.966	0.344
Acute kidney injury	7.6	8.3	15.1	30.0	44.4	100	<0.001	<0.001	0.004	0.026	0.363
Permanent pacemaker implantation	15.9	14.8	12.1	10.0	5.5	0	0.098	0.141	0.601	0.627	0.862
Major bleeding	9.8	10.2	18.2	6.1	11.1	0	0.198	0.838	0.508	0.506	0.478
New onset atrial fibrillation	2.8	4.9	13.3	40.8	42.3	0	<0.001	<0.001	–	0.884	0.459
Paravalvular leak	9.8	11.1	6.1	0	5.5	0	<0.001	<0.001	0.083	0.021	0.318

SAVR, surgical aortic valve replacement; STS, Society of Thoracic Surgeons; TAVI, transcatheter aortic valve implantation; TIA, transient ischemic attack.

## Discussion

The results of this single-center, retrospective cohort study showed that the type of procedural approach for AS—whether TAVI or SAVR—did not affect in-hospital, 30-day, or 1-year mortality. Therefore, the anticipated advantages of using a less invasive technique did not appear to influence early or mid-term outcome. Rates of PPI and paravalvular leak were more frequent in the TAVI group, whereas new onset AF and acute kidney injury were more common after SAVR. After stratification by STS score, there were no differences in survival between groups, whereas a statistically significant difference was found in low-risk and intermediate-risk groups in terms of moderate or severe paravalvular leak, in favor of SAVR, and in acute kidney injury and new-onset AF, in favor of TAVI.

With the development of TAVI over the past two decades and its introduction into routine clinical practice, it was inevitable to ask whether TAVI is the best treatment for all octogenarians (16, 17). The results of the Placement of Aortic Transcatheter Valves (PARTNER)-1 trial—the first prospective randomized study comparing TAVI, best medical therapy, and surgical therapy in high-risk patients—were published in 2011. Rates of all-cause death at 1-year were similar in the TAVI and SAVR groups, and were superior to best medical therapy (7). PARTNER 2A and Surgical Replacement and Transcatheter Aortic Valve Implantation (SURTAVI), both of which were designed to compare outcomes in intermediate-risk patients, also displayed non-significant differences between TAVI and SAVR in the 1-year non-hierarchical composite of all-cause death or disabling stroke (8, 9). Recent trials focusing on low-risk patients—PARTNER 3 and Medtronic Evolut Transcatheter Aortic Valve Replacement in Low Risk Patients (EVOLUT LRT)—opened new doors for TAVI and established the role of this approach in all risk groups. However, caution is needed when applying the findings from RCTs, which are subject to strict enrollment criteria, to the spectrum of patients with AS treated in routine clinical practice (18).

Our primary outcome, 1-year survival, was not influenced by the type of procedure. Recent studies focusing solely on octogenarians are scarce and show similar outcomes for death (17). Other reports use different composite outcomes, making direct comparisons difficult (19–22). A meta-analysis by Witberg et al. (23) suggested a trend toward reduced rates of 1-year death with TAVI in RCTs in low-risk populations, whereas observational studies with PSM in the same meta-analysis showed a trend toward increased rates of death with TAVI. In our study, there was no statistically significant difference in hospital or 30-day mortality in the entire cohort or in the low-risk or intermediate-risk groups between TAVI and SAVR. Comparisons in the high-risk group were not possible because of the small number of patients who underwent SAVR. After performing PSM in our population there was a statistically significant difference between the groups in terms of hospital stay, in hospital and 30-day mortality. Mollmann et al. (24) reported lower in-hospital mortality with TAVI in an all-comer AS population treated in Germany. However, a systematic review and meta-analysis by Moss et al. (17) in octogenarians showed no differences in 30-day mortality between TAVI and SAVR.

The rate of PPI in the present study was higher in the TAVI group, in which 15.0% of patients developed postprocedural conduction disturbances. The 9.3% of patients requiring PPI after SAVR is similar to the rate reported in other studies in octogenarians (17, 25) as well as in major TAVI studies (7–11). Interestingly, even though more than half of our patients in the surgical arm had a sutureless self-expanding (Perceval or Enable) or balloon expandable (Intuity) valve implanted, the incidence of PPI was low (26–28). Data regarding the effect of new PPI after TAVI on mortality and morbidity are inconclusive (29). When expanding the indication for TAVI to the younger population, it is important to consider that PPI-related complications are more frequent in younger patients (30).

Postoperative moderate-to-severe paravalvular leak was present in approximately 10% of TAVI patients and in only 0.8% of SAVR patients. Compared to previous RCTs (7–10) our TAVI population presented with relatively high incidence of postoperative moderate-severe paravalvular leak. This could in part be attributed to the fact that we only had access to early echocardiographic findings, with the ultrasound of the heart performed during hospitalization or before the discharge. At least a 30 day follow-up ultrasound would be necessary to properly evaluate our cohort. The PARTNER trial showed that increased severity of paravalvular leak is associated with higher 2-year mortality (31). Hagar et al. (32), however, found no association between paravalvular leak and 1-year mortality in a slightly younger population (mean age 74 years).

Neurological complications in our study were in line with the PARTNER-2 and SURTAVI results for the SAVR group (3% for cerebrovascular stroke or TIA), whereas results in the TAVI group were similar to those of the PARTNER 3 and EVOLUT LRT results, with a much lower incidence (0.7% of cerebrovascular stroke or TIA) despite a higher incidence of patient-related risk factors (33). It is difficult to explain this difference between TAVI and SAVR groups. We could attribute the incidence of neurological complications to unmeasured patient and periprocedural characteristics or operator skill and experience.

New onset AF (within 30 days of the procedure) was significantly more frequent in the SAVR group and is similar to the rate reported in RCTs (7–11) and retrospective studies (34–36). It is important to consider that almost half of the patients in the TAVI group had AF preoperatively. A number of studies evaluated possible risk factors and preventive measures. Older age and moderate-to-severe left atrial enlargement were the only two consistent independent factors of prolonged postoperative AF (34). Mathew et al. (37) showed that each consecutive decade was associated with a 75% increase in the risk of developing postoperative AF. Postoperative pericardial effusion is frequently observed after surgery, and experimental studies indicate that even a small amount of effusion may trigger AF due to mechanical compression of the atria, local inflammation, and oxidative stress (38).

Acute kidney injury was present in 32.7% of our SAVR patients. In studies conducted before the use of common diagnostic criteria [RIFLE (Risk, Injury, Failure, Loss of kidney function, and End-stage kidney disease) or AKIN (Acute Kidney Injury Network)], the reported incidence of acute kidney injury was lower than in our study (39, 40), but later studies showed similar findings, with acute kidney injury present in approximately one-third of patients after SAVR (41–44). An important finding was that even small changes in serum creatinine concentration are associated with adverse outcomes (45). In SAVR, the specific reason is cardiopulmonary bypass that causes inflammation and hemodilution and is accompanied by periods of low pressure and flow rates. The duration of cardiopulmonary bypass and aortic cross-clamp time were linked to the development of postoperative acute kidney injury (46). In our study, the average aortic cross clamp time was 47.0 ± 17.7 min, with a 50% higher occurrence of acute kidney injury in the SAVR group with an aortic cross-clamp time exceeding 50 min. Contrast exposure, embolization, hemodynamic instability, and access route play important roles in acute kidney injury after TAVI. Transapical access, compared with transfemoral access, was shown to be an independent predictor of acute kidney injury following TAVI (45), which was also seen in our study.

Major or life-threatening bleeding occurred in 11.0% of TAVI patients and in 6.7% of surgical patients, but the difference was not statistically significant. Octogenarians are a more vulnerable population because of their advanced age, comorbidities and frailty, independent of the STS score (47, 48), and a higher rate of bleeding complications was expected. The low rate of bleeding in SAVR group could be a consequence of using minimally invasive techniques (76.6% of patients underwent minimally invasive SAVR), and the high rate of bleeding complications in the TAVI group could be due to the large size of the delivery systems used.

Stratification by STS score into three groups showed that quite a sizeable number of patients in TAVI group were low risk. With rapid expansion of TAVI volumes, the risk profile of patients treated in everyday practice has been lower than those included in RCTs, which are the basis for practice guidelines (23). Our results showed no statistically significant differences between specific risk groups (TAVI vs. SAVR) in 30-day or 1-year mortality. Further, the stratification showed the expected significant difference between TAVI and SAVR in low- and intermediate-risk groups in occurrence of acute kidney injury, paravalvular leak, and new onset AF. Analysis of the high-risk group was not possible because it included only 2 SAVR patients.

The promising results from TAVI trials have fundamentally changed the way we treat octogenarians. It is important to take into consideration that not all patients, due to their advanced age, are automatically high risk (49). STS-PROM and STS/American College of Cardiology in-hospital mortality scores are superior to EuroSCORE I, EuroSCORE II, and the German AV Score (50). Despite more than 10 years of clinical experience with TAVI, a reliable risk score model that includes frailty is not yet available. Certain octogenarians with a low STS score may benefit more from a surgical procedure and avoid the possibility of paravalvular leak, which affects overall survival. A reliable risk score would help us recognize such patients and tailor their treatment individually. Treatment options should remain open, and regular revision of “real-life” results should ensure that we can offer patients the best treatment option. Detailed explanation of possible complications and balanced information regarding outcome after each procedure should be given to obtain informed patient consent prior to the choice of procedure.

### Study limitations

This single-center study was observational, non-randomized, and retrospective. Some perioperative variables were not recorded, which may explain the differences between groups. Patients in the SAVR group did not routinely have a computed tomography scan before the procedure. The two techniques are not directly comparable, because the decision-making may have taken into consideration variables not included in the scoring system and in our database.

## Conclusion

This retrospective study involving an octogenarian “real-life” population treated for isolated severe AS provides insights into our decision-making and shows that similar short- and mid-term results were obtained with both TAVI and SAVR in low- and intermediate-risk (STS score) groups. We showed that the type of procedural approach did not significantly affect 1-year mortality. The expected advantage of a less invasive technique did not influence early outcome, with similar rates of in-hospital mortality and length of hospital stay in both groups. PPI and paravalvular leak were more frequent in the TAVI group, whereas new onset AF and acute kidney injury were more common in the SAVR group. In light of our findings, the treatment strategy in each institution should be adopted according to its own outcome data and facilities.

## Data availability statement

The raw data supporting the conclusions of this article will be made available by the authors, without undue reservation.

## Ethics statement

The studies involving human participants were reviewed and approved by the Ethical committee of Republic of Slovenia. Written informed consent for participation was not required for this study in accordance with the national legislation and the institutional requirements.

## Author contributions

TK and MB: conception and design of the study. TK and AK: data collection. TK, NL, AK, DŠ, ZF, and MB: analysis and interpretation. TK, NL, DŠ, ZF, and MB: drafting and critical review of the article. All authors have read and approved the final manuscript.
